# Publisher Correction: Neural attentional-filter mechanisms of listening success in middle-aged and older individuals

**DOI:** 10.1038/s41467-021-26494-3

**Published:** 2021-10-19

**Authors:** Sarah Tune, Mohsen Alavash, Lorenz Fiedler, Jonas Obleser

**Affiliations:** 1 Department of Psychology, University of Lübeck, Lübeck, Germany; 2 Center for Brain, Behavior, and Metabolism, University of Lübeck, Lübeck, Germany; 3 Present Address: Eriksholm Research Centre, Snekkersten, Denmark

**Keywords:** Attention, Language

Correction to: *Nature Communications* 10.1038/s41467-021-24771-9, published online 26 July 2021.

In the original version of this Article, Fig. 2 was inadvertently omitted in the PDF version of the Article. This has now been corrected in the PDF version of the Article.

