# Supplementary material for: Neural attentional-filter mechanisms of listening success in middle-aged and older individuals
Source: Nat Commun. 2021 Jul 26;12:4533. doi: 10.1038/s41467-021-24771-9 (PMC8313676; doi:10.1038/s41467-021-24771-9)
Supplement: Supplementary file 3 — Reporting summary [file 41467_2021_24771_MOESM3_ESM.pdf]

nature research

Corresponding author(s): Sarah Tune, Jonas Obleser

Last updated by author(s): Jun 12, 2021

Reporting Summary

Nature Research wishes to improve the reproducibility of the work that we publish. This form provides structure for consistency and transparency in reporting. For further information on Nature Research policies, see our Editorial Policies and the Editorial Policy Checklist.

Statistics

For all statistical analyses, confirm that the following items are present in the figure legend, table legend, main text, or Methods section.

n/a	Confirmed	
☐	☒	The exact sample size (*n*) for each experimental group/condition, given as a discrete number and unit of measurement
☐	☒	A statement on whether measurements were taken from distinct samples or whether the same sample was measured repeatedly
☐	☒	The statistical test(s) used AND whether they are one- or two-sided<br>*Only common tests should be described solely by name; describe more complex techniques in the Methods section.*
☐	☒	A description of all covariates tested
☐	☒	A description of any assumptions or corrections, such as tests of normality and adjustment for multiple comparisons
☐	☒	A full description of the statistical parameters including central tendency (e.g. means) or other basic estimates (e.g. regression coefficient) AND variation (e.g. standard deviation) or associated estimates of uncertainty (e.g. confidence intervals)
☐	☒	For null hypothesis testing, the test statistic (e.g. *F*, *t*, *r*) with confidence intervals, effect sizes, degrees of freedom and *P* value noted<br>*Give P values as exact values whenever suitable.*
☒	☐	For Bayesian analysis, information on the choice of priors and Markov chain Monte Carlo settings
☐	☒	For hierarchical and complex designs, identification of the appropriate level for tests and full reporting of outcomes
☐	☒	Estimates of effect sizes (e.g. Cohen's *d*, Pearson's *r*), indicating how they were calculated

Our web collection on statistics for biologists contains articles on many of the points above.

Software and code

Policy information about availability of computer code

Data collection	PsychoPy Standalone v2.0, BrainVision Recorder
Data analysis	R v.3.6.1 (packages lme4 v.1.1-23, sjPlot v2.8.5); MATLAB 2016b, mTRF toolbox (v1.5) for MATLAB, Fieldtrip toolbox (v20170428) for MATLAB, Functional parcellation (Glasser et al. 2016, Nature), EEGlab (version 14_1_1b), Cortical mesh in accordance with Human Connectome Project (HCP) standard atlas template was generated by means of HCP Workbench (version 1.5; wb_command using FieldTrip script ft_postfreesurferscript.sh), FreeSurfer (ver 6.0)<br>Code for all analyses supporting this work is publicly available in the study's Open Science Framework repository (https://osf.io/nfv9e/).

For manuscripts utilizing custom algorithms or software that are central to the research but not yet described in published literature, software must be made available to editors and reviewers. We strongly encourage code deposition in a community repository (e.g. GitHub). See the Nature Research guidelines for submitting code & software for further information.

Data

Policy information about availability of data

All manuscripts must include a data availability statement. This statement should provide the following information, where applicable:

- Accession codes, unique identifiers, or web links for publicly available datasets
- A list of figures that have associated raw data
- A description of any restrictions on data availability

The complete neural and behavioral data required to reproduce the analyses supporting this work, as well as the auditory stimuli used in this study are publicly available in the study's Open Science Framework repository (https://osf.io/nfv9e/). Source data are provided with this paper.

Field-specific reporting

Please select the one below that is the best fit for your research. If you are not sure, read the appropriate sections before making your selection.

- [x] Life sciences
- [ ] Behavioural & social sciences
- [ ] Ecological, evolutionary & environmental sciences

For a reference copy of the document with all sections, see nature.com/documents/nr-reporting-summary-flat.pdf

Life sciences study design

All studies must disclose on these points even when the disclosure is negative.

Sample size	A total of N=155 participants were included in the final analysis. All participants are part of an ongoing large-scale longitudinal research project for which a representative sample of aging individuals between 40–80 years of age was recruited. No power analyses were computed as the overall sample size far exeeded the typical sample sizes in relevant prior studies.
Data exclusions	Three participants dropped out of the study prior to EEG recording and an additional 9 participants were excluded from analyses after EEG recording: three due to incidental findings after structural MR acquisition, and six due to technical problems during EEG recording or overall poor EEG data quality. As part of the initial screening process 17 participants were excluded prior to EEG recording due to non-age-related hearing loss or medical history.
Replication	The experiment involved the presentation of 240 trials (60 per condition) per participant. Results per condition are shown at the level of the group and the individual.
Randomization	Presentation of individual trials was randomized for each participant. Presentation of individual sentences to the left and right ear was counterbalanced across participants following randomized assignment; assignment of probed ear was counterbalanced across trials within participants.
Blinding	Blinding was not relevant to the present study as only one group of participants was included who took part in all conditions.

Reporting for specific materials, systems and methods

We require information from authors about some types of materials, experimental systems and methods used in many studies. Here, indicate whether each material, system or method listed is relevant to your study. If you are not sure if a list item applies to your research, read the appropriate section before selecting a response.

Materials & experimental systems

n/a	Involved in the study	
x		Antibodies
x		Eukaryotic cell lines
x		Palaeontology and archaeology
x		Animals and other organisms
	x	Human research participants
x		Clinical data
x		Dual use research of concern

Methods

n/a	Involved in the study	
x		ChIP-seq
x		Flow cytometry
	x	MRI-based neuroimaging

Human research participants

Policy information about studies involving human research participants

Population characteristics	Healthy middle-aged and older adults (39–80 years old) were included in the study. All participants were right-handed native Germany speakers. They had normal or corrected-to-normal vision, did not report any neurological, psychiatric, or other disorders and were screened for mild cognitive impairment using the German version of the 6-Item Cognitive Impairment Test. Only participants with normal hearing or age-adequate mild-to-moderate hearing loss were included.
Recruitment	Participants were recruited from the Institute of Psychology human subjects database (recruited mainly via advertisements in the local newspaper and following science outreach activities such a public talks). We aimed at recruiting a representative sample of healthy aging adults from various demographic background yet an educational selection bias cannot be fully precluded. Such a bias might lead to overestimating the absolute performance in challenging listening situation but should leave the relative effects at the neural or behavioural level unaffected.
Ethics oversight	Participants gave written informed consent and received financial compensation (8€ per hour). Procedures were approved by the ethics committee of the University of Lübeck and were in accordance with the Declaration of Helsinki.

Note that full information on the approval of the study protocol must also be provided in the manuscript.

Magnetic resonance imaging

Experimental design

Design type	Does not apply.
Design specifications	Does not apply.
Behavioral performance measures	Does not apply.

Acquisition

Imaging type(s)	structural
Field strength	Siemens MAGNETOM Skyra 3T
Sequence & imaging parameters	Structural images were collected using a magnetization prepared rapid gradient echo (MP-RAGE) sequence [TR =1900 ms; TE =2.44 ms; FA =9°; 1-mm isotropic voxel; 192 sagittal slices].
Area of acquisition	whole-brain acquisition

Diffusion MRI ☐ Used ☒ Not used

Preprocessing

Preprocessing software	Fieldtrip toolbox (v20170428) for MATLAB (ft_volumereslice: 256x256x256 voxels, 1mm resolution; ft_volumerealign: ctf coordinate system; ft_volumesegment: 'brain', 'skull', 'scalp'; ft_prepare_mesh: [3k 2k 1k] verticies; ft_headmodel: 'dipoli', ft_transform_geometry: MNI to CTF), FreeSurfer (ver 6.0): recons-all).
Normalization	Linear normalization as implemented in Fieldtrip toolbox (v20170428) for MATLAB (ft_volumerealign: cfg.coord = 'spm'; ft_volumereslice: 256x256x256 voxels, 1mm resolution).
Normalization template	MNI (SPM12 default)
Noise and artifact removal	Does not apply.
Volume censoring	Does not apply.

Statistical modeling & inference

Model type and settings	Does not apply.
Effect(s) tested	Does not apply.

Specify type of analysis: ☐ Whole brain ☐ ROI-based ☐ Both

Statistic type for inference (See Eklund et al. 2016)	Does not apply.
Correction	Does not apply.

Models & analysis

n/a	Involved in the study
☒	☐ Functional and/or effective connectivity
☒	☐ Graph analysis
☒	☐ Multivariate modeling or predictive analysis
